# Nurses’ experiences of blood sample collection from children: a qualitative study from Swedish paediatric hospital care

**DOI:** 10.1186/s12912-022-00840-2

**Published:** 2022-03-15

**Authors:** Henrik Hjelmgren, Britt-Marie Ygge, Björn Nordlund, Nina Andersson

**Affiliations:** 1 Astrid Lindgren’s Children’s Hospital, Karolinska University Hospital, Stockholm, Sweden; 2 Department of Women’s and Children’s Health, Karolinska Institute, 171 77 Stockholm, Sweden

**Keywords:** Nurses’ experiences/ perspective, Thematic analysis, Focus group, Blood sampling procedure, Children

## Abstract

**Background:**

Nurses play an active role in supporting the children with the blood sampling experience. Unfortunately, the blood sampling collection procedure is often affected by pre-analytical errors, leading to consequences such as delayed diagnosis as well as repeated sampling. Moreover, children state that needle procedures are the worst experience of their hospital stay. The nurses’ experiences of errors occurring during blood sample collection is unknown. Therefore, the aim of this study therefore was to describe paediatric nurses’ experiences of blood sampling collections from children.

**Method:**

We used a qualitative study design with a (reflexive) thematic analysis (TA) method described by Braun and Clarke. Three focus group interviews were conducted, with 19 nurses collected by purposeful sampling from Sweden working at two different paediatric hospitals, focusing on their experiences of the blood sample collection procedure.

**Results:**

From the three focus group interviews we analysed patterns and meanings of the following main theme *Paediatric blood sampling is a challenge for the nurses* and the four subthemes *Nurses’ feelings of frustration with unsuccessful samplings*, *Nurses believe in team work*, *Venous blood sampling was experienced as the best option*, and *Nurses’ thoughts and needs regarding skills development in paediatric blood sampling*.

**Conclusion:**

The narrative results of this study illustrate that nurses working in paediatric hospital care face a big challenge in blood sampling collection from children. The nurses felt frustrated due to unsuccessful blood samplings and frequently could not understand why pre-analytical errors occurred. Nevertheless, they felt strengthened by colleagues in their team and shared feelings of responsibility to help each other with this complex procedure. The implications of this study are that paediatric hospital care needs to focus on improving guidelines for and increasing competence in blood sampling children and helping nurses to understand why samplings may be unsuccessful and how this can be avoided.

## Background

Nurses on children’s wards play an active role in helping the children with the experience and also in reducing potential adverse effects of blood sampling collection [1]. Blood sampling collection is crucial to determining the correct diagnosis and providing children with treatment. The blood sample process can be divided into three phases: pre-analytical, analytical and post-analytical phases, where errors in pre-analytical phases represent up to 70% [2]. The pre-analytical phase includes prescription of a blood sample test, preparation and execution of blood sample collection, as well as safe transportation of the blood sample to the laboratory where the analytical phase starts [3]. Unfortunately, the pre-analytical phase in blood sampling collection from children has been found to be commonly affected by pre-analytical errors, which could risk patient safety and comfort [4]. Possible consequences of pre-analytical errors are many: delayed treatment, wrong diagnosis, repeated sampling and increased costs [5–7]. The literature states that the most common pre-analytical errors are haemolysed sample, unfilled or inappropriate sample, clotted sample, wrong container, patient identification or transport problems [8]. Inside Swedish paediatric hospitals, blood sampling is mainly performed by nurses without support from laboratory personnel [9]. Blood sampling collection from children is a difficult and complex procedure, meaning that the procedure requires the hospital staff to receive special training and pay extra attention in order to achieve good patient care and good quality blood sampling [10, 11].

Hospitalized children list blood sampling and needle procedures as one of the worst experiences of their hospital stay [12, 13]. They have difficulties complying with blood sampling collection because it often leads to pain and stressful situations [11, 14].

The interaction in the hospital between the child and the paediatric nurse is also complex. By listening to the child’s and the parents’ proposals, pain and discomfort can be limited during invasive procedures [15]. Anaesthetic nurses have described that knowledge about children’s fears and their stages of development are necessary for an optimal caring situation [16].

Recently, on 1^st^ January 2020, children’s rights became law (2018:1197) in Sweden, meaning that the rights of the child shall be taken into account in all deliberations and assessments made in decision-making processes in cases and matters concerning children. This is pertinent to the role of nurses working in Swedish paediatric hospitals, who must have knowledge, skills and specific competence concerning blood sampling procedures, which include preparations and support adapted to each individual child’s development [17]. In paediatric hospitals in Sweden, nurses perform both venous- and capillary blood sampling methods on hospitalized children. In general, the existing national and international guidelines for blood sampling procedures specific to paediatric hospital care are thinly designed. The Swedish Handbook of Health Care [18] is mostly focused on adult care, as are the European Federation of Clinical Chemistry and Laboratory Medicine(EFLM) venous guidelines [19] and the American Clinical and Laboratory Standards Institute(CLSI) guidelines [20, 21]. CLSI are not open access but they include venous and capillary guidelines focusing on adult care with some extra information for children. The World Health Organization(WHO) phlebotomy guidelines [22] provide some structured information but omit a number of children-specific topics, for example, how to approach children’s different developmental stages, ages and anatomical challenges, and how to avoid pre-analytical errors.

There is a lack of evidence surrounding paediatric nurses’ experiences of the blood sampling procedure with children and of their experiences of the errors occurring in the pre-analytical phase. Their experiences are important for aligned interventions to be created in the future, with tailored educational activities for reducing pre-analytical errors.

### Aim

To describe nurses’ experiences of blood sampling procedures with hospitalized children in a paediatric hospital context.

## Method

### Study design

We performed a qualitative study with a thematic content analysis approach [23, 24], using focus group interviews with nurses from two different academic paediatric hospitals in Stockholm, Sweden.

### Data collection

We conducted three focus group interviews with registered nurses that perform blood sampling collections from children. Focus group interviews are particularly good when it comes to describing people’s experiences and attitudes. The object of focus group interviews is to receive data which is high-quality and in a social context where the participants can reflect on their own views in relation to others [25]. The Consolidated criteria for reporting qualitative research (COREQ) checklist was used in this study to ensure a comprehensive report [26].

### Sampling

In this study, purposeful sampling was carried out to find different heterogeneity groups characterised by nurses in different stages of their career. A purposeful sampling also means you want to identify informative participants to generate rich informative data and in-depth information about the particular phenomena you want to investigate [25]. The participants were approached by email or face-to-face and given information by the nurse managers.

A flexible interview guide was first conducted by the main author (HH) and then discussed and revised by the co-authors (NA, BMY). Each interview started with open-ended questions and followed by probing questions to elicit more elaborative answers [25]. NA conducted the first interview. NA and BMY were present for the second interview, while HH and NA were present for the final one. The interviews lasted between 39–58 min and were audio recorded. The interviews were carried out between September-December 2019.

### Participants

The focus groups included registered paediatric nurses with different levels of experience and education. Table 1 describes their age and clinical background. A written consent document was handed out and signed at the time of the interviews.

**Table 1:** Demographics of participants

Interviews number	Participants (n)	Age (Mean)	Work place	Registered Nurse(RN) /Master degree Nurse(MSN)	Length of work experience (mean)
Nurses Group 1	9	26	4 wards	RN	10.6 months
Nurses Group 2	6	33	2 wards	MSN	6.7 years
Nurses Group 3	4	28	2 wards	RN	7 months

### Setting

This study included participants from two paediatric hospitals in Stockholm, Sweden, caring for a wide range of conditions. We included two hospitals to be able to cover a wider range views and experiences. One hospital is a tertiary hospital with oncology, surgical, medical and intensive care units, while the second hospital is smaller regional hospital, with two medical wards. The nurses came from different wards in the hospitals, as well as from their emergency department. Approximately 220,000 children and adolescents from birth to 18 years of age are living in the area covered by Stockholm County Council. The interviews were conducted in comfortable and nicely spaced conference rooms close to the clinic, which aimed to create a relaxed and peaceful environment for the interviews.

### Data analysis

A qualitative (reflexive) thematic analysis (TA) was chosen as a theoretical framework for this study, and the applied method of analysis was used for the transcribed interviews. Research with qualitative design gives access to patient perspectives and offers a wide range of methods to investigate whatever interest, including interaction between health care provider and patient or health organisation politics [27]. Due to there being only fragmented previous knowledge of our research question [23, 24], we converged the TA with an “inductive” approach, as described by Clark & Braun (2006) and recently clarified and discussed by the same (2020). The organisation of data went through the six described phases, including transcribing, making notes and coding, and creating themes composed from code patterns and meaning of data [28]. The audio recordings were transcript verbatim by the two authors HH and NA. The software Microsoft Excel and Microsoft Word were used to organize the data. The authors (HH, NA, BMY) familiarized themselves with the data and then discussed the initial patterns after the coding. The first author is a specialist paediatric nurse with preunderstanding of the blood sampling process and the affected errors. The three other authors were previously clinical active in the tertiary paediatric hospital, but now work with research or educational activities.

**Fig. 1:**
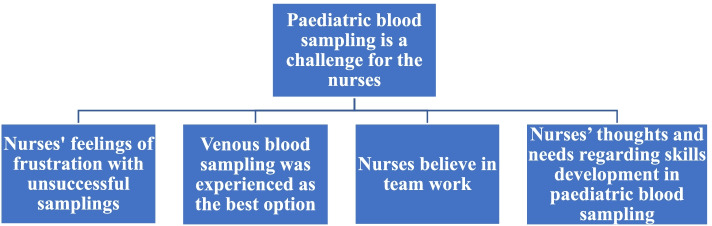
Thematic map

The analysing process went back and forth and as themes were discussed, a thematic map was created and revised and refined throughout the process (Fig. 1).

### Ethical considerations

All registered nurses who were asked volunteered to participate in this study. Participants were given oral and written information about the study beforehand and told they had the right to withdraw at any time. All participants were assured confidentiality and their identities were coded and concealed from all parties apart from the first author (HH). The study was approved by the Regional Ethical Review Board in Stockholm (2015/206–31/4).

## Results

From the transcribed data of the focus group interviews we found patterns and meaning for an overall main theme with four subthemes that all relates to the main theme. The overall main theme was “Paediatric blood sampling is a challenge for the nurses”. The four subthemes was: “Nurses’ feelings of frustration with unsuccessful samplings”, “Nurses believe in team work”, “Venous blood sampling was experienced as the best option”, and “Nurses’ thoughts and needs in regard to developing skills in paediatric blood sampling”. These themes are presented as a narrative, with illustrative quotes describing the participants’ experiences of blood sampling procedure with children.

### Main theme: Paediatric blood sampling is a challenge for the nurses

The nurses believed it was more of a challenging process to take blood samples from children than from adults. They viewed the sampling process as more complex and complicated. It was not only the puncture itself which was more difficult, but also the whole situation surrounding it.

“Yes, there’s a huge difference when you’re working on adults but with a child it could be a process that takes a whole morning just to get near them.” (Nurses Group 2).

The nurses felt that they first had to build the children’s confidence to ensure the blood sampling procedure went smoothly. Building confidence was important because the nurses knew the procedure might be repeated several times during the child’s hospital visit. Feeling stressed and wanting to perform high quality care but being unsuccessful was another challenging aspect. The informants were often experienced in adult care, where they felt self-confident, but this changed when they started working in the paediatric hospital.

The participants experienced ethical and moral conflicts related to blood sampling which were challenging, especially when they had to do something against the child’s will. The nurses felt it was inhumane to repeat the blood sampling procedure multiple times. However, the nurses sometimes felt they had no other choice because the sample was key to the diagnosis and proper management of the child.

“How many times is it humane to needle a child? That’s always the ethical question that’s difficult”. (Nurses Group 1).

Working with the whole family was another challenging aspect of the blood sampling procedure. The children’s parents could often interfere and make the nurse’s relationship with the child difficult. For example, the parents could say things that made the nurses feel angry or frustrated.

“No, it’s not really ideal when a parent says: “here comes the mean nurse to jab you (laughing) and it’ll hurt”. (Nurses Group 2).

Other times the parents wanted to be optimistic for the child, telling them the blood sampling procedure would “go fine with no pain” and that it would be just “one jab”, when the nurses knew this was not true. This made the nurses feel insecure and could lead to lack of confidence and trust between the nurse and child. The nurses often had to deal with the anxiety and fears of both the child and their parents.

The nurses also expressed awareness that children of certain ages or with special needs could present an extra challenge to the procedure. For example, hospitalized children with severe acute or chronic conditions were a category that made the process even harder. The nurses reflected on the fact that the sampling process was time consuming and planning their work could be a challenge. Additionally, the participants felt that moments such as preparations could be challenging but were very important.

“Sometimes when you go in to take samples it all goes really smoothly and that’s good but quite often you have to give yourself time to ensure it goes well, the next time and the time after that”. (Nurses Group 2).

The participants occasionally had poor self-confidence, which could be challenging to cope with. It was easier to help a colleague with a difficult patient than to succeed with their own patients.

### Sub theme 1. Nurses’ feelings of frustration with unsuccessful samplings

The nurses experienced frustration around several aspects of unsuccessful blood sampling. Although they might have thought a sampling went well, the hospital lab results recorded in the medical journal reported otherwise, due to pre-analytical errors, such as a clot or haemolysis. The nurses were frustrated that the laboratory never explained what went wrong, merely stating that the sample could not be analysed. Many times the nurses had put all their effort and fighting spirit into the procedure and when the results came back reporting errors, this caused sadness, frustration and anger.

“You get very angry and I called the lab and asked why it was like this and then they had no real answer so then you get really angry.” (Nurses Group 1).

Sometimes the nurses felt that the “machines”, especially the bedside analyses, were working against them. They also perceived that certain blood analyses like the blood gas test, INR coagulation test and amniotic fluid analysis test were difficult to fulfil, which illustrated a lack of knowledge around these aspects. Often the nurses felt they had problems getting too little or too much blood in the collecting micro tubes. The participants thought they had the correct amount and were then perplexed when the laboratory responded by reporting the result to be an “unfilled sample”.

“And then on occasion, when I’ve taken the same sample from a child three times and all three have coagulated each time, and when I really know I turned everything and warmed it up and did everything, that from here on, now there’s something strange – something spooky about it”. (Nurses Group 3).

When the nurses could not believe it was their own mistake, they tried to give other explanations for why sampling was unsuccessful. If not the machines, could it be the quality of materials or even sloppy laboratory staff? The nurses’ ambiguity and uncertainty seemed to nudge them into a blaming culture.

“Sometimes I’ve got the feeling that they just drop the samples and then they (the lab) have the cheek not to report it. Everything went perfectly, and then the haemolysis, you just go what?! Oh no!” (Nurses Group 2).

The nurses thought it was better to send the collected blood samples to the laboratory, even though they were uncertain they had been successful. Often they defended this by expressing concerns for the children in that they did not want them to have too many punctures or suffer from hospital-acquired anaemia. The nurses felt the doctors were unaware of how many blood samples they prescribed or the risks of anaemia, which led the nurses to difficult prioritisations.

“What priorities so we can try to take them if you’ve jabbed (the child) once or twice to get the first samples then you want to chance it and send them, sometimes you can write ‘very difficult patient to needle’ so sometimes I do that…it’s kind of the best we can get”. (Nurses Group 1).

There were occasionally situations when help was needed but not given from elsewhere and communication was poor. The nurses felt frustrated, bringing their concerns for the child in focus.

“Yes, but it feels disappointing. I’m not asking help for my sake – I can push the needle in ten times but it’s for the child’s sake, isn’t it – so you don’t damage the vessels.” (Nurses Group 1).

During the interviews the nurses frequently expressed uncertainty about how to handle the samples or about what and why pre-analytical errors occurred for their specific sampling.

### Subtheme 2: Nurses believe in team work

As demanding and complex as the blood sampling can be, the nurses said they felt the presence of facilitators could ease the procedure. They believed a supportive team and good communication would contribute to successful sampling and that having at least two to three colleagues on hand during the procedure was a good idea, helping them to make use of distraction methods and manage the samples effectively.

“…better if there’s more of you, not just for distraction but also so you have someone who can hand you things, stand and turn tubes”. (Nurses Group 3).

The nurses felt that both physical and psychological support from each other were essential for a qualitative sampling procedure. They mentioned that they were able to ask for help and, if necessary, they could spontaneously change blood sampling method or even who was in charge of the needling.

“…but it’s also thanks to having such great back-up and support from our colleagues that no-one ever sighs when you ask for help, they’re very positive and cheerful”. (Nurses Group 1).

During the interviews, the nurses described experiences which showed that they had a deeper understanding of the child’s needs and comfort. They viewed good communication in the team with parents as important and often of benefit in the situation.

“But they (the parents) are really important in it going well. Because if they start getting stressed about things or say stuff that has nothing to do with it or whatever, it can go belly up because of it”. (Nurses Group 2).

The experienced nurses often investigated the child’s condition first and could feel when sampling was unnecessary, prompting them to ask the clinicians to rethink the ordering of blood samples in order to reduce the number of punctures.

### Subtheme 3: Venous blood sampling was experienced as the best option

Another subtheme was “Venous blood sampling was experienced as the best option”. The different sampling methods discussed were capillary- and venous blood sampling. During these discussions, the participants interacted and asked each other questions about which method they preferred. Pros and cons were discussed and venous sampling was mainly viewed as the best option by all focus groups. The nurses said venous sampling could benefit the blood flow and increased the chances of capturing good quality blood specimens.

“If you’ve learnt venous it’s easier than capillary, better flow and it increases the chances of getting good samples”. (Nurses Group 1).

One of the participants was positively surprised when she performed a venous puncture on an infant, which led to her suddenly having collected six micro tubes without problem, the tricky part instead being that she had too much blood to handle.

Choosing a method according to the individual child in front of them was described as important. The nurses felt that they had many factors to think about, such as the child’s age and developmental stage. One nurse stated that the choice of sampling method could minimize pain for child.

“It depends on the child. I often think it hurts more if you sample the finger.” (Nurses Group 2).

In regard to capillary sampling, one participant explained it as a minor procedure, while another stated it was seldom used. However, capillary sampling was possibly a better choice if a child had special needs, for example, spasticity. Getting the right amount of blood for the ordered analyses was another aspect discussed in regard to choosing the right method. For the nurses, the fewer punctures they made, the better.

“And then maybe it only takes one needle in the vein instead of three capillary, yes, to get enough blood”. (Nurses Group 3).

Blood sampling is a multifaceted task and the nurses described their skills in planning for the best interests of the child. If the child needed a periphery cannula, the nurses tried to collect blood specimens at the same time from the same cannula. The nurses said that when patients had a new existing cannula, it made things more pleasant for both them and the child, as they could continuously withdraw samples without punctures and pain for the child. It was necessary to plan your procedure accordingly, due to the few available chances nurses have for collecting blood. Another factor the nurses discussed was the limited number of visible and small vessels a small child has.

“ …Not just to have fewer needlings but also because you might not have that many chances to take, in the first instance, venous samples – they’ve kind of got a certain number of vessels that are even possible to try on”. (Nurses Group 2).

### Subtheme 4: Nurses’ thoughts and needs regarding skills development in paediatric blood sampling

The last subtheme was “Nurses’ thoughts and needs regarding skills development in paediatric blood sampling”. The nurses described how it felt to lack knowledge of paediatric care and mentioned what they had missed out on during their introduction programs or even university nursing education. Some participants also felt important information had been omitted about the differences between paediatric and adult blood sampling procedures and that university nursing programs had failed to mention this in their training. The nurses felt that learning by doing and by observing colleagues was the main way nurses embraced knowledge. They reflected on their own competence related to blood sampling children, stating that they often tried to “join in” with more experienced colleagues conducting blood sampling procedures to discover “tips and tricks” for their own use. The first focus group stated that, in future, they would like an annual continuous professional development course in sampling techniques.

“Everyone should get trained…just like getting CPR once a year, you can have needle training once a year.” (Nurses Group 1).

Another aspect was that some nurses lacked education concerning preparations and choosing the right blood sampling method, as well as knowledge about the amount of blood that could be taken from the children.

“The thinking around sampling and perhaps a bit more on which ones I can actually take from capillaries and which ones have to be venous, so that’s what I wish I had in my training.” (Nurses Group 3).

Some nurses said that simulation training was a bonus during their paediatric nurse introduction, but that it did not feel like reality and could not simulate real clinical situations they experienced. The nurses were eager to learn more and wanted to improve their skills but did not know how and when it could done.

“It’s hard to practise all situations on a simulation doll or things like that, also that there are things that have to be done in order to improve”. (Nurses Group 2).

## Discussion

This study sought to describe the nurses’ experiences of the blood sampling procedure with children. From the study data, we described one major theme and four subthemes, which relates to successful or unsuccessful blood sampling procedures.

The overall main theme *Paediatric blood sampling is a challenge for the nurses* illustrates that nurses working in paediatric hospital care face a big challenge with the blood sampling collection procedure for children. The four subthemes: *Nurses’ feelings of frustration with unsuccessful samplings*, *Nurses believe in team work*, *Venous blood sampling was experienced as the best option and Nurses’ thoughts and needs regarding skills development in paediatric blood sampling* describe the nurses’ diverse experiences concerning blood sampling in children. To the best of our knowledge these are new findings and not published elsewhere.

The overall main theme of our study was *Paediatric blood sampling is a challenge for the nurses*. The nurses felt that the whole procedure was very different from sampling adults, and this highlights their holistic approach and concerns for the hospitalized child. Our results also highlighted challenges in coping with parents, children with special needs and the nurses’ own self-confidence during the procedure. An American study investigating phlebotomist experiences [29] described anxious patients and parents as a primary problem in relation to several aspects of blood sampling in children. Parents can often unwittingly transfer their own fears and anxiety to their children, something nurses must often be well-prepared to manage. In our study, the nurses also faced ethical dilemmas, for example, the number of punctures required and sometimes, restraining the child against its will. Children in hospital are a vulnerable group and any restraining should be minimized by good planning and adjusted pain relief methods by the healthcare personnel with focus on the patients dignity and privacy [30]. This highlights the wide range of issues a paediatric nurse must cope with when executing blood sampling. Nurses in other clinical contexts have also described conflicting emotions when they deviate from instructions, have to hold patients still or when parents interfere during their children’s blood sampling [31]. To assure the safety of the child and protect its rights, nurses must use their clinical judgement in each situation and for each individual child so that they can appropriately tailor the best preparations and interventions, before, during and after the procedure [32]. The United Nations children’s convention from 1989 confirmed the rights of children to be supported, protected and respected, and for them to participate with their dignity recognised. Although these obligations are clearly stated, an Italian study of paediatric nurses’ responses found that hospitalized children’s rights are still not implemented fully [33]. Another study has also pointed out that the organization must recognise that extra time and a high level of clinical competence and resources are needed for advanced paediatric care [16].

In this study, *Nurses’ feelings of frustration with unsuccessful samplings* was one of the most interesting subthemes we analysed. Unsuccessful samplings with pre-analytical errors, such as clots, unfilled sample and haemolysis were something the nurses often experienced. They could not believe it when the blood sample came back from the laboratory as a failed analysis, which created stress, anger and frustration. This dilemma illuminates the knowledge gap and the grey zone between nursing care and laboratory medicine in paediatric hospital care. In other needle-related procedures, such as venous access in children, nurses have reported increased job satisfaction, performance as well as improved relationship with patient and family when they learned more about pain management for the procedure [34]. If nurses gained more knowledge on how to avoid pre-analytical errors it might lead to the same results where nurses can reduce their stress and frustration. In the aforementioned blood sampling guidelines, there is seldom any detailed instruction on how to avoid possible pre-analytical errors, something which could have helped the paediatric nurses. None of the participants in the focus groups mentioned reporting unsuccessful samplings as incidents. This was recently demonstrated in another study, where nurses described that they lacked time, routines and guidance for incident reporting unsuccessful sampling [35]. Unsuccessful samplings also meant the nurses needed to take time from other important care. Nurses in other contexts have been found to lack knowledge about pre-analytical errors but are eager to learn [36], which was also the case with the nurses in our study. The participants mentioned several aspects regarding pre-analytical problems, for example, technical issues, communication with the laboratory, as well as not knowing why a clot or other errors had occurred.

The nurses in our study were at liberty to choose the appropriate sampling method. Our analysis revealed they felt that *Venous blood sampling was experienced as the best option,* and this was another theme in this study. There was a difference between inexperienced and experienced nurses in the way they discussed sampling method. The more experienced nurses expressed deeper concerns about the child’s needs and comfort. This shows how important it is to motivate the younger nurses to take part in continuous professional development to increase their knowledge [37]. Overall, venous sampling was more often considered the first and best choice because blood flow would be better and more blood could be collected. This has been demonstrated to be more successful in Hjelmgren et al. 2021 [38]. The nurses could have been helped further if the guidelines had been more specific about when to use which sampling method for age and developmental stages and analyses. Interestingly, the safety of both personnel and child were seldom discussed, even though the different sampling methods incur different risks for both patient and staff and are described in WHO phlebotomy guidelines [22]. The methods were more often discussed in terms of whether the procedure was complicated or not. Capillary blood sampling was seen as an option when the child had few visible veins or had special needs. However, there are medical devices that visualise the veins, which could benefit venous withdrawal of blood in children [39].

The subtheme *Nurses believe in team work* illustrates that the nurses searched for ways to cope with the complex blood sampling procedure and did so by communicating with parents and gathering colleagues in the team. Other studies have described the importance of promoting the safety of hospitalized children as a challenge shared by both parents and the health personnel team [40]. In paediatric care the nurses should be responsive and aim to listen to the child’s previous experience and to be supportive in the needle-related procedures [41]. In our study, having an assistant on hand to stabilize the arm or distract the patient was viewed as important, which is consistent with the recommendations in CLSI venepuncture guidelines [20]. The nurses in our study viewed it as important for both capillary and venous sampling.

The nurses sometimes felt doctors ordered a lot of unnecessary sampling, creating unwanted suffering for the children. They were also worried that all this sampling could lead to risk of hospital-acquired anaemia. If the child’s condition changed for the better, the nurses often questioned whether sampling was necessary. Literature has described an overutilization of laboratory testing in hospital settings and that resting is often of no clinical importance [42]. The frequent overdraw is a documented potential risk of hospital-acquired anaemia [43], which confirms the nurses’ concerns and should lead to doctors and health organisations improving care and communication concerning this aspect.

*Nurses’ thoughts and needs regarding skills development in paediatric blood sampling* evolved into our last subtheme. The participants had many thoughts and ideas on educational aspects, such as being given a good introduction to paediatric blood sampling, practical tips and repeated training in order for them to feel comfortable with the procedure. Previous research has shown that simulation learning could be a strategy which could create competency-based education, with holistic and context-dependent content for nurse educators to use [44]. The nurses in this study had mainly acquired knowledge from college or learning by doing. These findings indicate that nurses lack knowledge and deeper understanding of handling specimens and avoiding pre-analytical errors, such as clots and haemolysis. Another study has described that by providing standardized training and education pre-analytical errors could be reduced [45]. The role of experts in laboratory medicine also has a part to play. This includes improved communication and provision of support and education to nurses and doctors, as well as the patients. The blood sampling process is a multilevel process that includes nursing care, laboratory medicine and medicine science, and this makes cooperation and communication for the patient’s best especially important [46].

### Strength & limitations

The COREQ guidelines were used as a help for reporting in this study and increased trustworthiness. COREQ contains a 32-item checklist including three domains: 1) research team and reflexivity, 2) study design and theoretical framework and, 3) analysis and findings [26]. We chose not to return the transcript (item 23) to the participants after the interviews, as we had not taken notes on who said what in the group.

To achieve credibility, it is crucial to find participants who are likely to have experiences of the phenomenon under study and are able to talk about it [47]. Our purposive sampling approach had this intention. This study used focus groups, which is a method that could give the researcher a certain depth of data and context. A strength of the study is that the method could also generate group effects and participant interaction, leading to a learning moment which is not possible during individual interviews [48, 49]. To attain trustworthiness, one of the authors (NA) was present at all focus group interviews. We also illustrated our findings and interpretations with quotes that give another aspect of transparency and trustworthiness in qualitative studies [26]. We believe we reached information power, as described in Malterud et al., 2016, after the three interviews including 19 participants. The more information the data holds, the lower number of participants needed [50].

In TA analysis, researcher subjectivity is seen as a resource which strengthens the reflexive engagement with the interpretation of data and theory [24]. The first author (HH) has deep knowledge and understanding of the given subject, which was a resource for the interpretation of the data. The first author was not present at the first two group interviews, as he knew some of the participants well. Self-awareness of the researcher is important to sustain credibility [51]. Even though we used a more inductive approach, it is meaningful to say that as researchers, we are not in a theoretical vacuum with no previous knowledge. The analysis process was therefore more like a continuum going back and forth [24].

These findings could be transferable to other similar contexts where paediatric nurses are in charge of the procedure, although we believe nurse assistants, phlebotomists or even doctors performing blood sampling on children could have similar experiences and gain knowledge by reading this paper. As stated in previous research, paediatric care nurses are already aware of comfort methods, such as distraction techniques and pain-reducing treatments [52]. Consequently, as a nurse you could execute a perfect distracted and comfort measured blood sampling procedure but still fail to get a satisfactory blood test result from the laboratory.

Further research needs to focus on improving the support and education of nurses performing blood sampling on children and in so doing, reducing the pre-analytical errors in paediatric hospital care. This may be done by implementing the latest evidence-based research in this field, along with a children’s rights approach. Increasing the knowledge, competence and skills of paediatric nurses is key to reducing the number of unsuccessful samplings in the future.

## Conclusion

The narrative results of this study illustrate that nurses working in paediatric hospital care face a big challenge in blood sampling children. The nurses felt frustrated due to unsuccessful blood samplings and could often not understand why pre-analytical errors occurred. They felt strengthened by colleagues in their team and shared feelings of responsibility to help each other with this complex procedure.

### Relevance to clinical practice

The collected qualitative data about the nurses’ experiences indicate a strong need to improve guidelines and increase nurses’ skills and knowledge concerning PAE through educational interventions. Such interventions would help nurses increase their competence as well as understand why sampling procedures fail and how this can be avoided.

## Data Availability

The data used and analysed during the current study are available from the corresponding author on reasonable request.

## Abbreviations

CLSI: Clinical and Laboratory Standards Institute
COREQ: The Consolidated criteria for reporting qualitative research (COREQ)
CPR: Cardiopulmonary resuscitation
EFLM: European Federation of Clinical Chemistry and Laboratory Medicine
TA: Thematic analysis
WHO: World Health Organization
